# The role of gut microbiota in predicting the weight loss following laparoscopic sleeve gastrectomy

**DOI:** 10.3389/fmicb.2025.1560368

**Published:** 2025-03-03

**Authors:** Jionghuang Chen, Bo Shen, Hongdan Shen, Linghua Zhu, Hong Yu, Yifan Tong, Weihua Yu

**Affiliations:** ^1^Department of General Surgery, Sir Run Run Shaw Hospital, School of Medicine, Zhejiang University, Hangzhou, China; ^2^Liver Regeneration and Metabolism Study Group, Sir Run Run Shaw Hospital, School of Medicine, Zhejiang University, Hangzhou, China; ^3^Provincial Key Laboratory of Precise Diagnosis and Treatment of Abdominal Infection, Sir Run Run Shaw Hospital, School of Medicine, Zhejiang University, Hangzhou, China; ^4^Nursing Department, Sir Run Run Shaw Hospital, School of Medicine, Zhejiang University, Hangzhou, China

**Keywords:** laparoscopic sleeve gastrectomy, gut microbiota, weight loss, efficiency, dynamically

## Abstract

**Background:**

Laparoscopic sleeve gastrectomy (LSG) has emerged as a highly effective intervention in the management of obesity. While there has been a recent surge in research exploring the relationship between obesity and gut microbiota, the association between gut microbiota and LSG remains relatively underexplored. This study aimed to investigate the relationship between gut microbiota and both early and later effects of LSG.

**Methods:**

In this retrospective study, clinical characteristics and preoperative fecal samples were collected from 52 individuals who underwent LSG. Using 16S rRNA gene sequencing, we compared the community composition, alpha diversity, and beta diversity of gut microbiota between patients who experienced efficient weight loss and those who did not. Additionally, comprehensive and correlation analyses were performed to identify potential associations between specific microbial taxa and LSG outcomes.

**Results:**

The abundances of gut microbiota in patients who experienced efficient weight loss and those who experienced general weight loss were comparable. However, the influence of gut microbiota on the efficacy of weight loss is dynamic. Specifically, the *Fusobacteriota* phylum significantly contributed to the early curative effects of LSG, while *Actinobacteriota* had a greater impact on the late curative effects. Additionally, *Proteobacteria* were found to mediate long-term efficacy through complex mechanisms.

**Conclusion:**

This study analyzed the preoperative gut microbiota signature to predict the efficacy of LSG, potentially offering valuable insights for clinical applications. Preoperative assessment of gut microbiota profiles could assist patients in their decision-making processes, particularly regarding the potential outcomes of LSG and the long-term impact of the procedure on their health.

## Background

Epidemiologically, obesity has been steadily rising. Obesity is associated with type 2 diabetes mellitus (T2DM), metabolic dysfunctions related to fatty liver disease (MASLD), and an increased risk of cardiovascular disease and certain cancers (Blüher, 2019). Laparoscopic Sleeve Gastrectomy (LSG) is a widely performed bariatric surgery that plays a crucial role in weight loss (Schwack et al., 2025; Maggard-Gibbons et al., 2013; Karami et al., 2021). By surgically removing approximately 60% of the greater curvature of the stomach, LSG is a surgical procedure that not only reduces stomach capacity but also reorganizes the endocrine function of the entire body, thereby improving glycolipid metabolism through the regulation of hormone levels and appetite suppression. This leads to significant decreases in body mass index (BMI), making LSG an increasingly popular option for managing obesity (Stefura et al., 2021; Engelmann et al., 2025; Thiel et al., 2025). While individuals undergoing these surgical interventions generally experience favorable health outcomes, the extent of these benefits varies substantially. Therefore, the ability to accurately predict the effects of weight loss prior to LSG is of paramount importance from a therapeutic perspective.

Given that the human gut microbiota plays a crucial role in metabolic processes, immune regulation, and overall health, it significantly shapes the biochemical composition of the diet and the host’s metabolism (Fan and Pedersen, 2021; Anhe et al., 2023; Zambrano et al., 2024). In terms of mechanism, the influence of gut micbiota on the body can occur through various pathways, including metabolic product alterations, brain-gut axis, and neuro-immune interactions, hormonal regulation, etc. (Li et al., 2025; Zhang et al., 2025). Previous studies have demonstrated that the composition of the gut microbiota is altered in obese individuals, which may affect energy metabolism, fat storage, and inflammatory responses. For instance, gut microbes in obese individuals are more likely to produce short-chain fatty acids, leading to increased energy absorption and further promoting fat accumulation (Xie et al., 2025; Boulange et al., 2016; Hu et al., 2024). Meanwhile, after undergoing LSG, patients often experience varying degrees of weight loss, and there are corresponding changes in their gut microbiota. These changes typically include an increase in *Bacteroidetes* and *Proteobacteria*, along with a decrease in *Firmicutes* (Georgiou et al., 2022; Lau et al., 2021; Chen et al., 2024).

Considering the alterations in the gut microbiota observed in obese patients and the variable weight loss outcomes following LSG, we hypothesized that the composition of the gut microbiota may influence the efficacy of LSG. Therefore, this study aimed to identify preoperative gut microbial signatures associated with weight loss by analyzing preoperative fecal samples through 16S rRNA sequencing. The findings provide insights into the potential interplay between gut microbiota and therapeutic outcomes in individuals undergoing LSG.

## Methods

### Study design

Between July and November 2021, a total of 56 patients who underwent LSG met the eligibility requirements at Sir Run Run Shaw Hospital, School of Medicine, Zhejiang University. Four patients were lost to follow-up and 52 patients were ultimately recruited. This study was approved by the Human Ethics Committee of Sir Run Run Shaw Hospital (No. SRRSH-2024-1011) and in accordance with the Declaration of Helsinki.

Clinical characteristics and preoperative fecal samples were collected, with the primary outcome defined as the weight loss effect at 1 month and at 3–6 months after LSG. Specifically, patients who completed follow-up 1 month after LSG were classified into the early LSG effect group, while those who completed follow-up at 3–6 months were classified into the later LSG effect group. The weight loss effect was calculated using the formula: (Post-BMI minus Pre-BMI)/ Pre-BMI/months × 100%. Given the cut-off points of weight loss effect in the early and later LSG effect arms were 12%/m and 7%/m, respectively, patients were divided into early general weight loss (EGWL, <12%/month) and early efficient weight loss (EEWL, ≥12%/month) groups, and later general weight loss (LGWL, <7%/month) and later efficient weight loss (LEWL, ≥7%/month) groups.

### Procedure of LSG

Using a 36 Fr bougie, the stomach transsection was initiated 4 cm from the pylorus. We kept stapling along the bougie up to the angle of His, keeping a distance of about 1 cm from the gastroesophageal junction to avoid constriction at the incisura angularis. Next, we reinforced the entire staple line with a continuous absorbable suture (3–0). A drainage tube was not routinely left, unless bleeding was suspected. Antibiotics was only given when infection was suspected.

### Sample collection and 16s rRNA sequencing

The fecal samples were obtained from patients 12–24 h before LSG, and then were collected using a sterile kit, and then stored in a −80°C refrigerator. Genomic DNA extraction from the samples utilized the DNeasy PowerSoil Pro kit (Qiagen, CA, USA), with DNA purity and concentration confirmed via agarose gel electrophoresis. Dilution of the extracted DNA to 1 ng/μL using sterile water preceded PCR amplification with specific primers (16S V4 region primers, 515F and 806R) containing barcodes. Amplification was performed using New England Biolabs’ Phusion® High-Fidelity PCR Master Mix with GC Buffer and high-fidelity enzymes. Library construction employed the TruSeq® DNA PCR-Free Sample Preparation Kit, with quantification done through Qubit and qPCR. After quality control, libraries underwent NovaSeq6000 sequencing. Demultiplexing of raw sequencing data was conducted based on barcode and PCR amplification primer sequences. Trimming of barcode and primer sequences, as well as read joining using FLASH software (V1.2.7), generated raw tags. Quality filtering via fastp (Version 0.23.1) produced high-quality Clean Tags. UCHIME Algorithm was applied to compare tags with the Silva database (16S/18S) and Unite Database (ITS) for chimera sequence detection, followed by removal. The remaining effective tags underwent denoising with the deblur module in QIIME2 software (Version QIIME2-202202) to obtain initial Amplicon Sequence Variants (ASVs). Species annotation was achieved using QIIME2 software.

### Data normalization and analysis

Normalization of ASV absolute abundance involved standardizing to a sequence number corresponding to the sample with the fewest sequences. All subsequent analyses of alpha diversity, beta diversity were conducted based on the normalized data output.

For comprehensive insight into community diversity, richness, and uniformity, alpha diversity, beta diversity was computed using indices in QIIME2: Chao1, Dominance, Shannon, Simpson, and Principal Co-ordinates Analysis (PCoA), Non-Metric Multi-Dimensional Scaling (NMDS). First, QIIME2 software was used to calculate the Unifrac distance, and R software was used to draw PCoA dimensionality reduction plots. Subsequently, use LefSe to complete species analysis of significant differences between group. Among them, LEfSe analysis and the default LDA score threshold were completed through LEfSe software. MetaStat analysis uses R software to test the differences between the two comparison groups at the six classification levels of phylum, class, order, family, genus and species and obtain *p* values.

Hierarchical clustering of samples was executed with the “pheatmap” package in R.^1^ To enhance trend visibility, bacterial abundance at the genus level was scrutinized. Clustering on the y-axis utilized the “Ward.d” function for Ward linkage distance calculation between samples, employing the “Euclidean” method. Visualization of clustering results was accomplished by generating a heatmap. When performing Spearman correlation analysis, first use the corr.test function of the psych package in R to calculate the Spearman correlation coefficient values of species and clinical factors and test their significance, and then use the pheatmap function in the pheatmap package for visualization.

### Statistical analysis

Continuous variables were expressed as medians with interquartile ranges and were compared using the Wilcoxon rank-sum test. Categorical data were represented as frequencies and percentages, and comparisons were made using the Pearson *χ*^2^ test or the Fisher exact test, depending on the suitability of the data. A two-sided *p*-value <0.05 was considered statistically significant. All data analyses were conducted using the SPSS statistical software package (version 22.0; IBM Corp).

## Results

**Figure 1:**
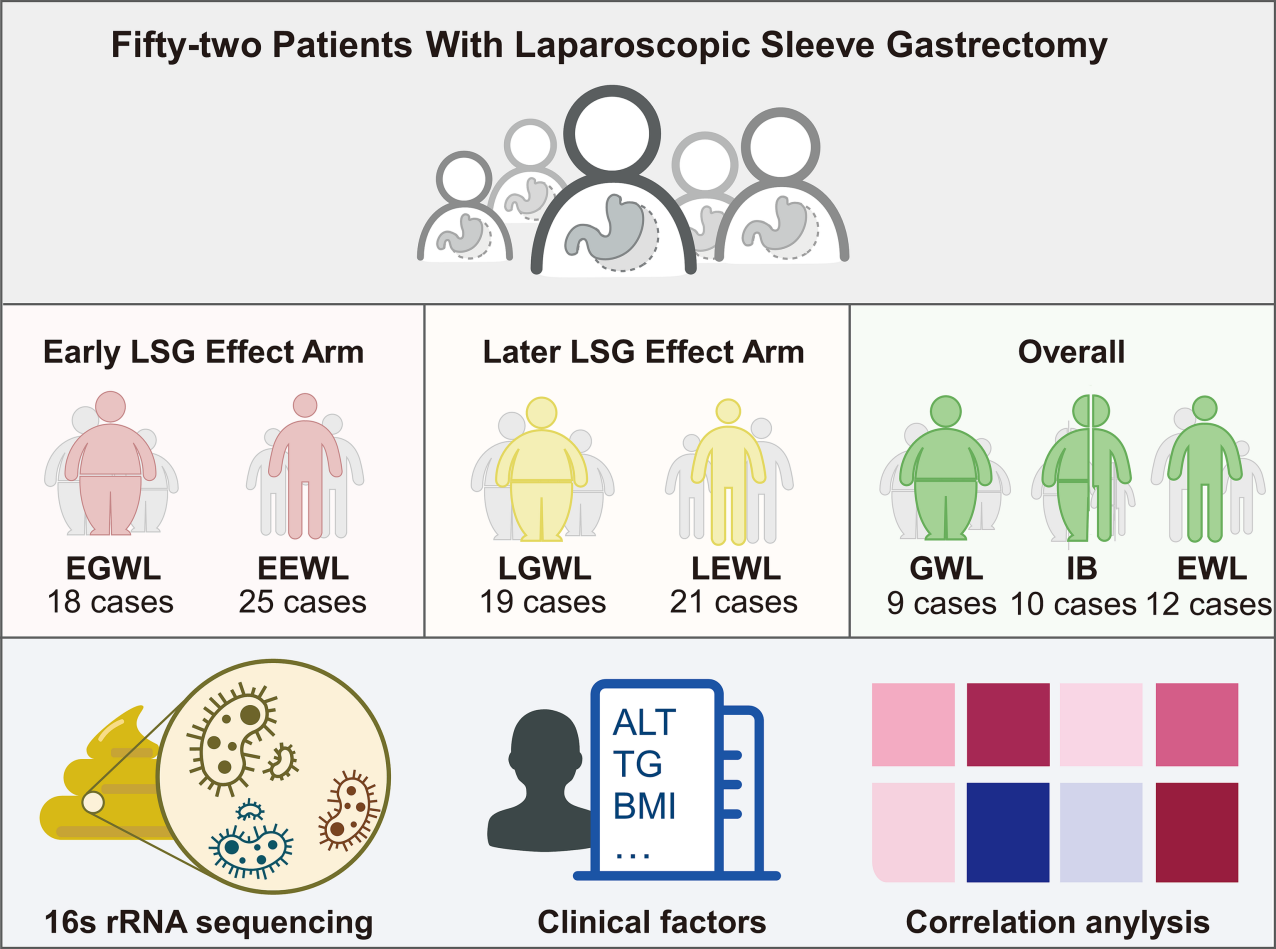
Flowchart of this study. A total of 52 patients after laparoscopic sleeve gastrectomy were enrolled. Then, the gut microbiota, clinical characteristics, and jointly analyses were performed.

The flowchart of this study was presented in Figure 1. Ultimately, 52 patients were enrolled in this study. Forty-three patients completed the early follow-up, 40 patients completed the later follow-up, and 31 patients completed both the early and later follow-ups. The clinical characteristics were summarized in Table 1.

**Table 1:** Clinical characteristics in the early and later effect arms.

	Early LSG effect arm (*n* = 43)			Later LSG effect arm (*n* = 40)		
	EEWL (*n* = 25)	EGWL (*n* = 18)	*p* value	LEWL (*n* = 21)	LGWL (*n* = 19)	*p* value
Pre-LSG BMI, kg/m^2^	36.3 (34.3–41.3)	38.4 (34.7–41.1)	0.331	36.6 (34.4–40.6)	38.1 (35.1–41.2)	0.503
Post-LSG BMI, kg/m^2^	31.4 (28.2–35.6)	34.1 (32.4–37.9)	0.015	28.0 (26.3–31.4)	30.1 (27.5–31.6)	0.236
ΔBMI, kg/m^2^	5.3 (4.7–6.1)	3.8 (3.0–4.3)	<0.001	9.1 (8.2–9.8)	9.0 (6.2–10.6)	0.851
Follow-up, months	1.0 (1.0–1.0)	1.0 (1.0–1.0)	1.000	3.0 (3.0–3.0)	4.0 (3.0–5.0)	<0.001
ΔBMI/month, kg/m^2^	5.3 (4.7–6.1)	3.8 (3.0–4.3)	<0.001	3.0 (2.7–3.3)	1.9 (1.8–2.2)	<0.001
ΔBMI/Pre-LSG BMI, %	13.9 (12.5–15.3)	9.5 (7.6–10.8)	<0.001	23.4 (21.9–25.3)	20.4 (16.6–27.7)	<0.001
ΔBMI/Pre-LSG BMI/month, %	13.9 (12.5–15.3)	9.5 (7.6–10.8)	<0.001	7.9 (7.3–8.4)	5.3 (5.0–5.9)	<0.001
Age, years	31.0 (26.5–34.0)	31.0 (28.0–35.5)	0.805	31.0 (25.5–36.5)	31.0 (28.0–37.0)	0.708
Gender (Male)	7 (28.0)	3 (16.7)	0.480*	4 (19.0)	6 (31.6)	0.473*
Smoking	6 (24.0)	4 (22.2)	1.000*	4 (19.0)	5 (26.3)	0.712*
Drinking	1 (4.0)	2 (11.1)	0.562*	1 (4.8)	3 (15.8)	0.331*
Hypertension	12 (48.0)	12 (66.7)	0.224	12 (57.1)	11 (57.8)	0.962
Fatty Liver	25 (100)	18 (100)	1.000*	20 (95.2)	19 (100)	1.000*
Diabetes	10 (40.0)	4 (22.2)	0.220	8 (38.1)	6 (31.6)	0.666
ALT, IU/L	49.0 (32.5–78.0)	31.0 (23.5–66.3)	0.183	31.0 (25.5–36.5)	33.0 (26.0–71.0)	0.979
AST, IU/L	33.0 (23.5–41.5)	25.5 (18.0–43.3)	0.307	30.0 (21.0–38.0)	27.0 (15.0–46.0)	0.708
FG, mmol/L	5.4 (5.1–7.5)	5.2 (4.9–6.4)	0.381	5.9 (5.2–7.8)	5.1 (4.3–6.6)	0.074
HbA1c	5.8 (5.5–6.6)	5.8 (5.4–6.1)	0.505	8.1 (5.5–8.2)	5.7 (5.4–6.1)	0.130
TG, mmol/L	2.1 (1.3–3.2)	1.9 (1.4–2.8)	0.375	1.9 (1.4–3.9)	2.0 (1.2–3.1)	0.893
LDLC, mmol/L	3.59 (3.07–4.24)	3.45 (2.90–4.09)	0.649	3.49 (3.22–3.87)	3.35 (2.84–4.00)	0.307
HDLC, mmol/L	1.00 (0.86–1.20)	1.03 (0.92–1.32)	0.445	1.08 (0.90–1.20)	1.03 (0.86–1.31)	0.957
FFA, umol/L	484.0 (334.0–560.0)	483.5 (386.8–663.3)	0.349	418.0 (322.5–584.0)	477.0 (383.0–649.0)	0.270

Data were presented as medians with interquartile ranges or numbers with percentages. LSG, Laparoscopic sleeve gastrectomy; EEWL, Early efficient weight loss; EGWL, Early general weight loss; LEWL, Later efficient weight loss; LGWL, Later general weight loss; BMI, Body mass index; ALT, Alanine transaminase; AST, Aspartate aminotransferase; FG, Fasting glucose; HbA1c, Glycosylated Hemoglobin, Type A1C; TG, Triglyceride; LDLC, Low-density lipoprotein cholesterol; HDLC, High-density lipoprotein cholesterol; FFA, Free fatty acid. The “*” represents the Fisher exact test.

**Figure 2:**
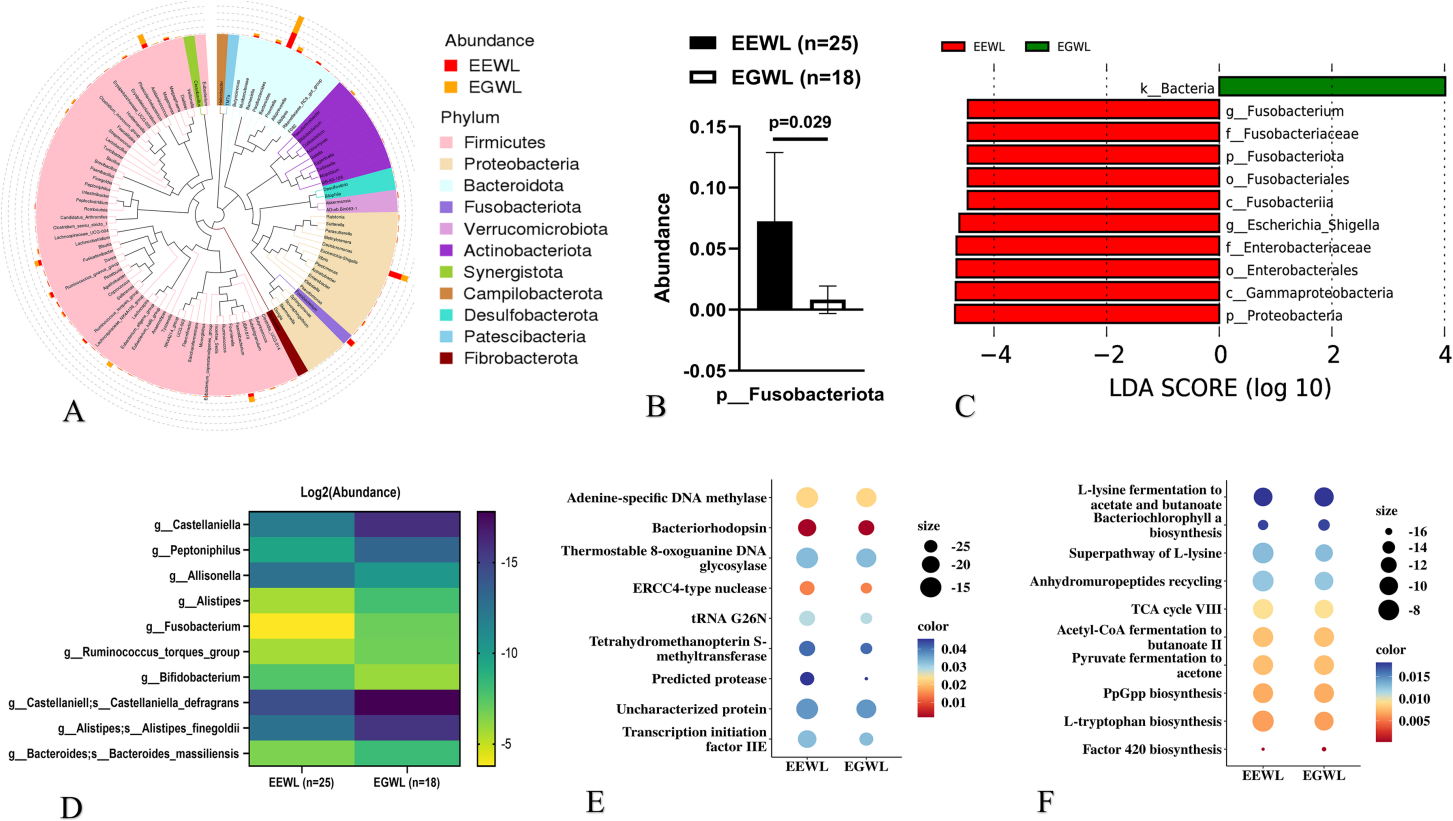
Analysis of gut microbiota in the early LSG effect arm. **(A)** The distribution of microbiota at phylum level. **(B)** The expression of Fusobacteriota in EGWL and EEWL groups. **(C)** LDA tree of LefSe analysis. **(D)** Significant difference microbiota at genus and species levels in EGWL and EEWL groups. **(E)** Picrust2 analyses by Cluster of Orthologous Groups (COG) databases. **(F)** Picrust2 analyses by Kyoto encyclopedia of genes and genomes (KEGG) metabolism databases. LSG, Laparoscopic sleeve gastrectomy; EEWL, Early efficient weight loss; EGWL, Early general weight loss.

In the early LSG effect arm (*n* = 43), 25 patients experienced effective weight loss (EEWL), while 18 patients experienced general weight loss (EGWL). The ΔBMI/Pre-LSG BMI/month(%) were 13.9 (12.5–15.3) and 9.5 (7.6–10.8) in the EEWL group and EGWL group (*p* < 0.001), respectively. Although the gut microbiota abundances in the EEWL and EGWL groups were comparable, there were minor variations in their distribution within the early effect arm (Figure 2A and Supplementary Figures 1A–C).

For example, the abundance of *Fusobacteriota* at the phylum level was significantly higher in the EEWL group (*p* = 0.029) (Figures 2B,C). However, *α*-diversity and *β*-diversity showed no significant differences between the EEWL and EGWL groups (Supplementary Table 1 and Supplementary Figure 1D). Further LefSe analysis revealed that patients in the EEWL group exhibited diverse microbiota signatures at various taxonomic levels (Figure 2D). Among these, the changes in the microbiota at the genus level are outlined in Figure 2E. Additionally, through PICRUSt2 analysis, which encompassed the Cluster of Orthologous Groups (COG) and Kyoto Encyclopedia of Genes and Genomes (KEGG) metabolism databases, we identified involvement in pathways such as adenine-specific DNA methylase and the L-lysine fermentation to acetate and butanoate pathways (Figures 2E,F).

**Figure 3:**
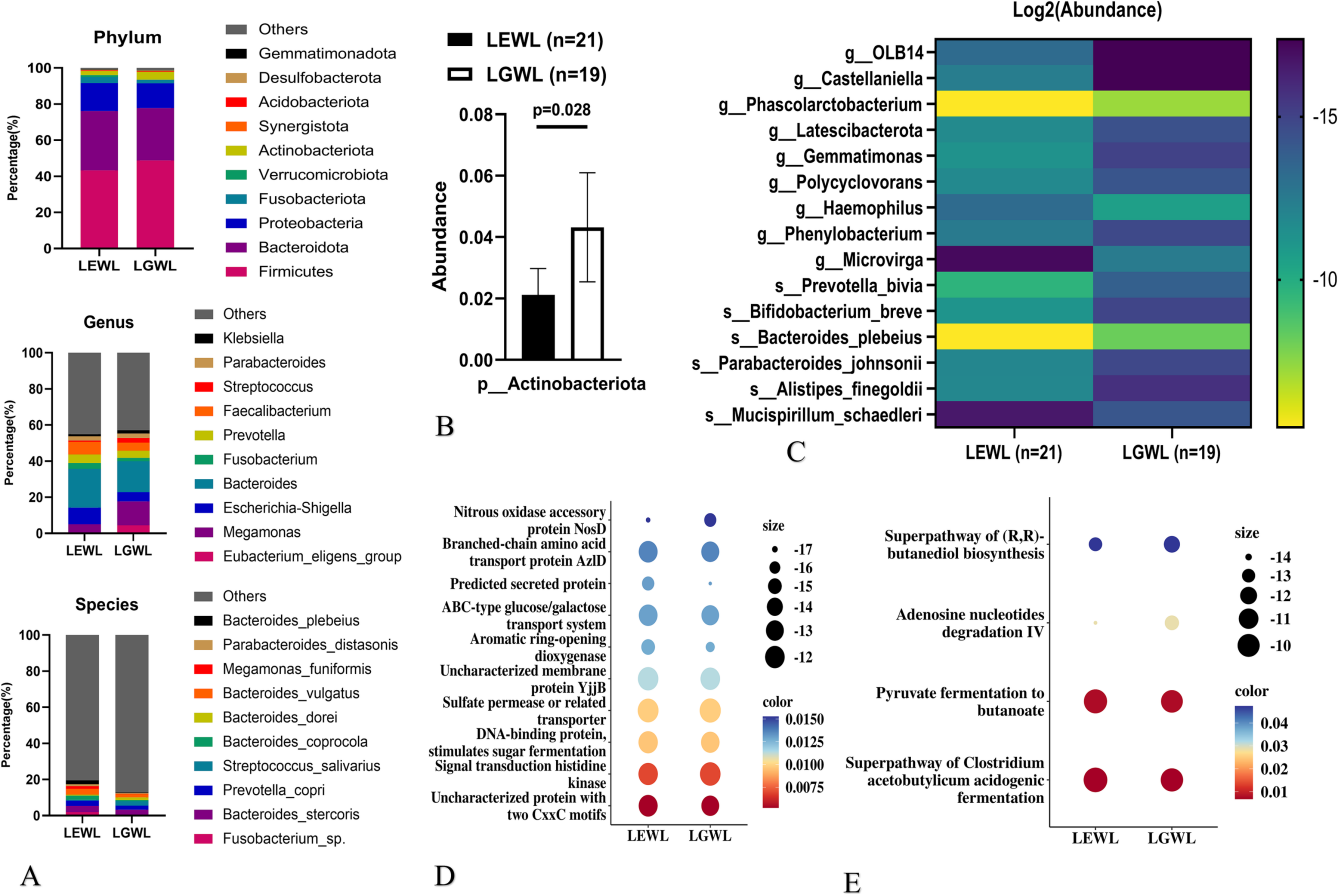
Analysis of gut microbiota in the later LSG effect arm. **(A)** The distribution of microbiota at phylum, genus and species levels. **(B)** The expression of *Actinobecteriota*. **(C)** LDA tree of LefSe analysis. **(D)** Picrust2 analyses by Cluster of Orthologous Groups (COG) databases. **(E)** Picrust2 analyses by Kyoto encyclopedia of genes and genomes (KEGG) metabolism databases. LSG, Laparoscopic sleeve gastrectomy; LEWL, Later efficient weight loss; LGWL, Later general weight loss.

In the later LSG effect arm (*n* = 40), 21 patients were in the efficient weight loss (LEWL) group, and 19 patients were in the general weight loss (LGWL) group. The ΔBMI/Pre-LSG BMI/month(%) values were 7.9 (7.3–8.4) for the LEWL group and 5.3 (5.0–5.9) for the LGWL group (*p* < 0.001), respectively. Overall, there was no significant difference in the abundance of gut microbiota between the LEWL and LGWL groups at various taxonomic levels (Figure 3A and Supplementary Figure 2A). The *α*-diversity and *β*-diversity were also comparable between the two groups (Supplementary Table 1 and Supplementary Figure 2B). However, the *Actinobacteriota* phylum showed a slight downregulation in the LEWL group (*p* = 0.028) (Figure 3B). In the LefSe analysis, *Megamonas* of the *Firmicutes* phylum and *Fusobacterium* of the *Fusobacteria* phylum were identified as being reduced in the LEWL group (Supplementary Figure 2C). Although not statistically significant, *Megamonas* and its species were predominantly found in LGWL patients at the genus and species levels (Figure 3C). Additionally, PICRUSt2 analysis revealed that genes related to ABC-type transport and pathways associated with *Clostridium acetobutylicum* were enriched (Figures 3D,E).

**Figure 4:**
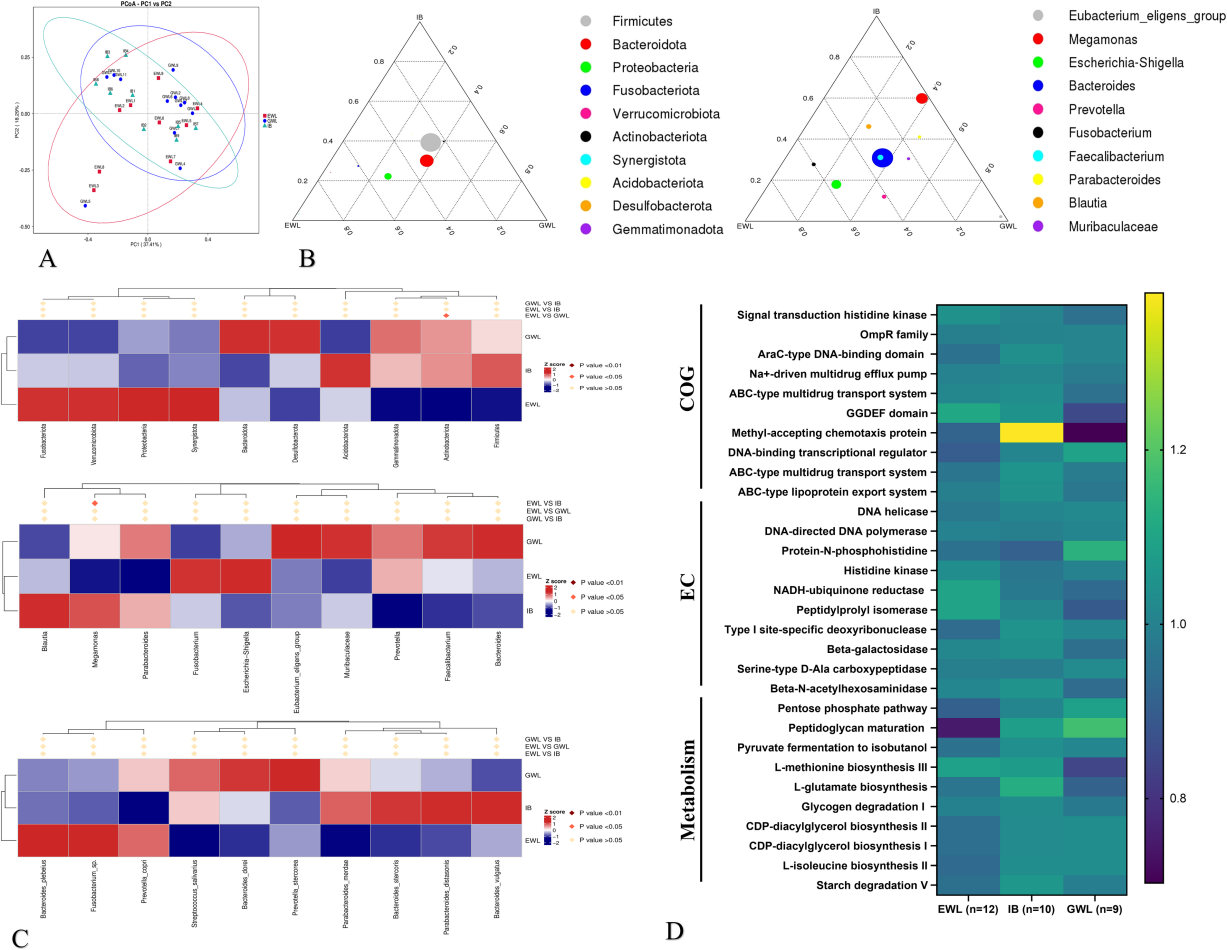
Comprehensive analysis of gut microbiota in patients with different LSG effects. **(A)** Beta diversity analysis. **(B)** Evolutionary trajectories (Left is early LSG effect arm and Right is later LSG effect arm). **(C)** MetaStat analysis at phylum, genus and species levels. **(D)** Functional pathway analysis by Cluster of Orthologous Groups (COG), enzyme commission nomenclature (EC) and Kyoto encyclopedia of genes and genomes (KEGG) databases. LSG, Laparoscopic sleeve gastrectomy; EWL, Efficient weight loss; GWL, General weight loss; IB, Inconsistent LSG effect between early and later phases.

To analyze the impact of preoperative microbiota composition on the overall effect of LSG, we combined the early and later effect arms and divided the patients into three groups (*n* = 31). Specifically, 12 patients exhibited both early and later efficient weight loss (EWL), 9 patients showed both early and later general weight loss (GWL), and 10 patients demonstrated inconsistent effects between the early and later stages (IB). The distribution at the phylum, genus, and species levels was shown in Supplementary Figures 3A–C. Generally, the microbiota distribution was similar between the GWL and IB groups (Figure 4A and Supplementary Figure 3D).

In the evolutionary trajectory analysis, *Proteobacteria* at the phylum level shifted from the GWL/IB to the EWL group during the early phase. During the later phase, *Escherichia-Shigella* exhibited similar trajectories (Figure 4B). Subsequent LefSe and MetaStat analyses identified *Megomonas* of the *Firmicutes* and *Actinobacteriota* as differentially expressed in the later LSG effect arm (Figure 4C). Finally, the PICRUSt2 analysis revealed that genes related to the ABC-type transport system and other metabolic pathways were enriched (Figure 4D).

**Figure 5:**
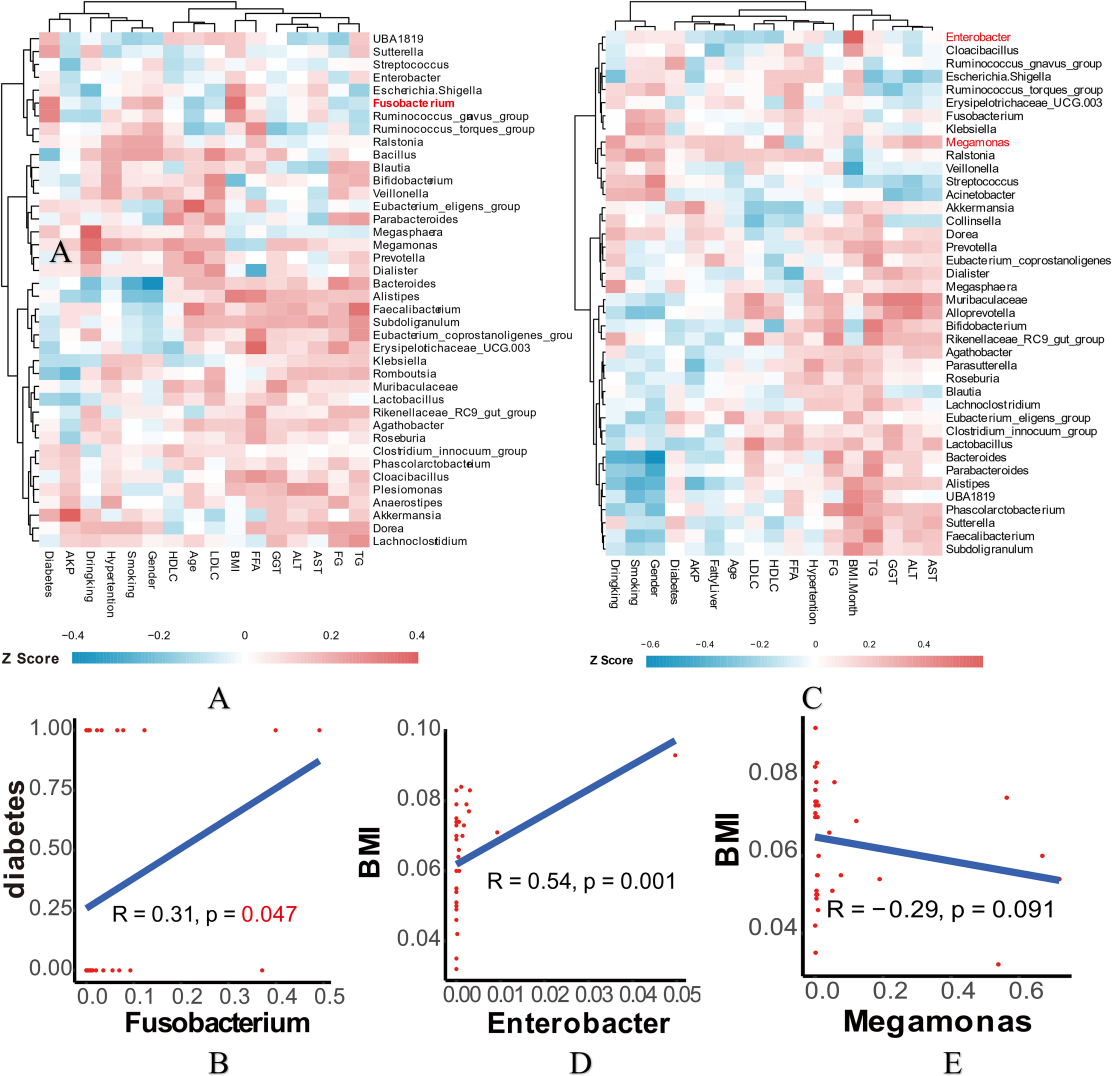
Correlation analysis in the early and later LSG effect arms. **(A)** Spearman correlation analysis heatmap in the early arm. **(B)** Correlation of *Fusobacterium* and diabetes. **(C)** Spearman correlation analysis heatmap in the later arm. **(D)** Correlation of *Enterobacter* and BMI. **(E)** Correlation of *Megamonas* and BMI. LSG, Laparoscopic sleeve gastrectomy; BMI, Body mass index.

As the *Firmicutes* is generally regarded as a beneficial partner in various diseases, our finding that *Megomonas* of *Firmicutes* was decreased in the EWL group prompted us to conduct a Spearman correlation analysis. This analysis aimed to examine the relationship between genus-level bacterial abundance and clinical indicators, providing further insight into how microbiota composition influences specific patient clinical outcomes (Figures 5A,C and Supplementary Figures 4A,B). In the early LSG effect arm, diabetes, rather than other indicators, exhibited a significant association with *Fusobacterium* (Figure 5B). In the later LSG effect arm, BMI was positively correlated with *Enterobacter* and negatively correlated with *Megamonas*, respectively (Figures 5D,E). Additional correlations were summarized in Supplementary Figures 4A,B. Taken together, our study highlights the dynamic interplay between gut microbiota and weight loss outcomes following LSG.

## Discussion

LSG is a surgical procedure used to treat morbid obesity, and it has been shown to improve the balance of gut microbiota and reduce chronic inflammation (Anhe et al., 2023; Zambrano et al., 2024). Compared to other bariatric surgeries, such as laparoscopic adjustable gastric banding (LAGB), LSG has distinct effects on gut microbiota (Zhang et al., 2025). For example, LSG has been demonstrated to increase colonic mucosal-associated invariant T cells (MAIT cells) while decreasing circulating regulatory T cells (Tregs) (Fukuda et al., 2022). These changes in the immune system and gut microbiota may explain the varying effects of LSG on T2DM and obesity (Kikuchi et al., 2018). However, there is currently a lack of detailed literature exploring the changes in gut microbiota among patients at different recovery stages. Understanding these changes could provide valuable insights into the mechanisms by which LSG exerts its therapeutic effects and help optimize patient outcomes. Further research is needed to comprehensively document the longitudinal changes in gut microbiota and their correlations with clinical outcomes in patients undergoing LSG.

In this study, preoperative gut microbiota dynamically regulates the effects of LSG. Specifically, early effect arm analysis revealed a higher abundance of *Fusobacterium* in patients experiencing efficient weight loss, whereas later effect arm analysis revealed an increased abundance of *Megomonas* and *Enterobacter* in those with general weight loss. *Megomonas* is a gut bacterium that has been demonstrated to be influenced by bariatric surgery, particularly in gastric bypass and LSG procedures. Previous studies have indicated that bariatric surgery alters gut microbiota composition, including changes in various genera such as *Megomonas* (Salman et al., 2021). This aligns with our findings, where higher levels of *Megomonas* were observed in patients with general weight loss. These results suggest that high levels of *Megomonas* may diminishes the therapeutic effects induced by LSG.

*Fusobacterium* has been studied in the context of bariatric surgery and gastric cancer. Previous research revealed that after Roux-en-Y gastric bypass surgery, *Fusobacterium varium* was transiently detected in the gut, while *Fusobacterium nucleatum* colonization correlated with decreased survival rates in *Helicobacter pylori*-positive gastric cancer patients (Coimbra et al., 2022; Palmisano et al., 2020; Hsieh et al., 2021; Stefura et al., 2022). Similarly, the administration of *Enterobacter cloacae* was found to increase adipose tissue hypertrophy and hepatic damage in high-fat diet-fed mice, reinforcing the link between *Enterobacter* and obesity identified in our research (Boehm et al., 2020; Keskitalo et al., 2018). In this study, elevated levels of *Fusobacterium* were associated with efficient weight loss at 1 month, though this does not necessarily imply long-term efficacy.

The present study has several limitations. First, it is confined to patients from a single hospital, and the sample size may not be sufficient for broader generalizability. Besides, patients were not monitored for an extended period following LSG, which limits our ability to assess long-term outcomes. Furthermore, we did not detect the stool samples at 1 month and 6 months post-LSG to further investigate the role of gut microbiota in weight loss and other postoperative changes. These limitations highlight areas for future research to enhance the generalizability and comprehensiveness of our findings.

In summary, this study suggests a preliminary association between gut microbiota and the prediction of the effects of LSG. Our findings indicate that preoperative gut microbiota dynamically regulates the effects of LSG. While further investigation is needed to clarify the role of gut microbiota in the context of LSG, this intriguing phenomenon may provide valuable insights for clinical applications and assist patients in their decision-making processes.

## Data Availability

The data reported in this paper have been deposited in the OMIX, China National Center for Bioinformation/Beijing Institute of Genomics, Chinese Academy of Sciences (https://ngdc.cncb.ac.cn/omix/, accession no. OMIX009130).

## Footnotes

^1^ https://CRAN.R-project.org/package=pheatmap

## Supplementary material

SUPPLEMENTARY FIGURE 1 The abundances of gut microbiota in the EEWL and EGWL groups. **(A–C)** The distribution of microbiota at phylum, genus and species levels. **(D)** Beta diversity analysis by Co-ordinates Analysis (PCoA), Non-Metric Multi-Dimensional Scaling (NMDS). LSG, Laparoscopic sleeve gastrectomy; EEWL, Early efficient weight loss; EGWL, Early general weight loss.

SUPPLEMENTARY FIGURE 2 The abundances of gut microbiota in the LEWL and LGWL groups. **(A)** The distribution of microbiota. **(B)** Beta diversity analysis by Co-ordinates Analysis (PCoA), Non-Metric Multi-Dimensional Scaling (NMDS). **(C)** LDA tree of LefSe analysis. LSG, Laparoscopic sleeve gastrectomy; LEWL, Later efficient weight loss; LGWL, Later general weight loss.

SUPPLEMENTARY FIGURE 3 The distribution in EWL, GWL and IB groups. **(A–C)** The distribution at phylum, genus and species levels in EWL, GWL and IB groups. **(D)** Beta diversity analysis by Non-Metric Multi-Dimensional Scaling (NMDS). LSG, Laparoscopic sleeve gastrectomy; EWL, efficient weight loss; GWL, general weight loss; IB, inconsistent effects between early and later stages.

SUPPLEMENTARY FIGURE 4 Spearman correlation analysis. **(A)** *Fusobacterium* and clinical characteristics correlation analysis. **(B)** *Enterobacter* and *Megamonas*, and clinical characteristics correlation analysis. LSG, Laparoscopic sleeve gastrectomy; BMI, Body mass index.
