# Supplementary figures and images for: The role of gut microbiota in predicting the weight loss following laparoscopic sleeve gastrectomy
Source: Front Microbiol. 2025 Mar 3;16:1560368. doi: 10.3389/fmicb.2025.1560368 (PMC11911518; doi:10.3389/fmicb.2025.1560368)

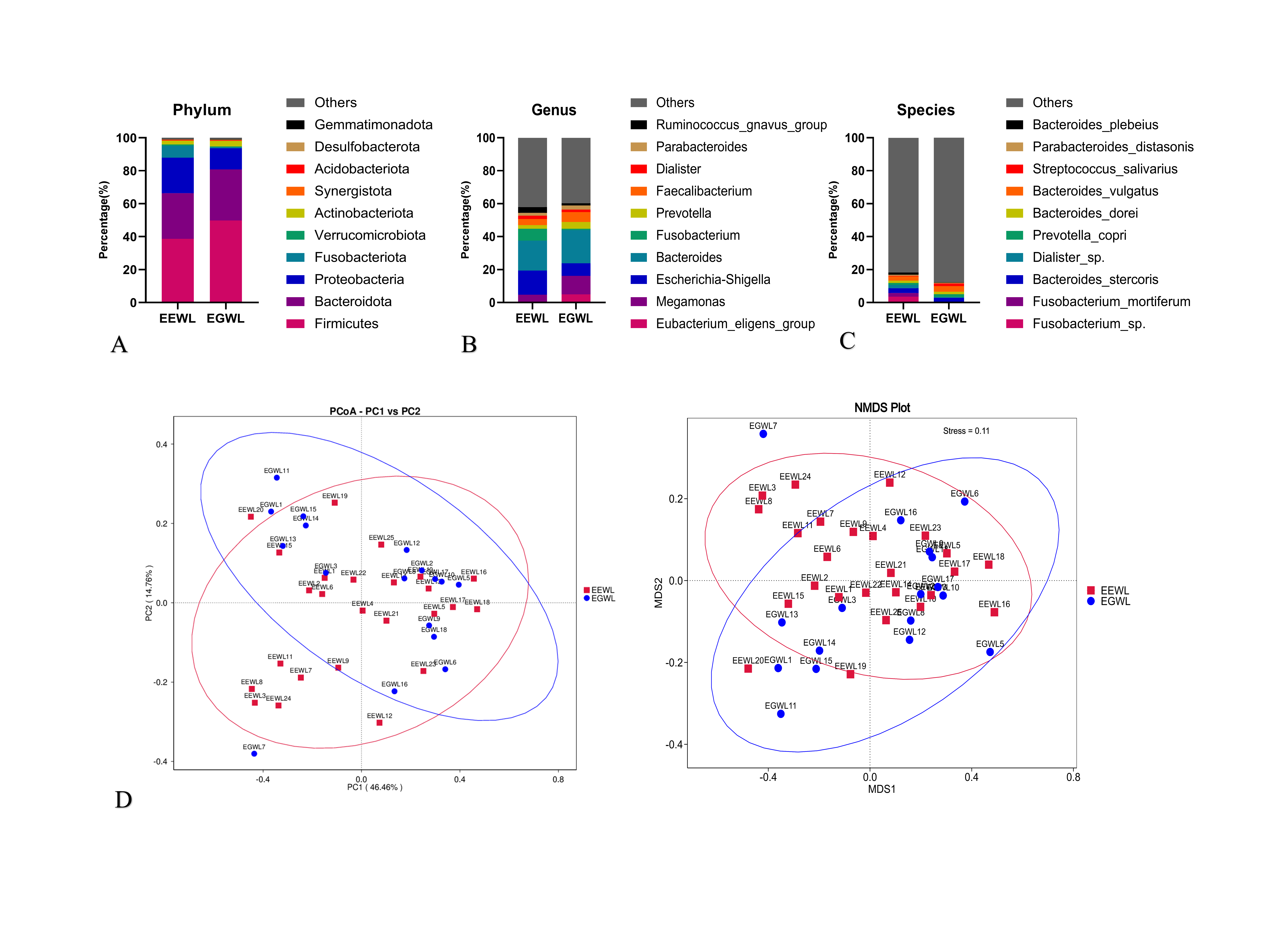

Supplement: SUPPLEMENTARY FIGURE 1 — The abundances of gut microbiota in the EEWL and EGWL groups. (A–C) The distribution of microbiota at phylum, genus and species levels. (D) Beta diversity analysis by Co-ordinates Analysis (PCoA), Non-Metric Multi-Dimensional Scaling (NMDS). LSG, Laparoscopic sleeve gastrectomy; EEWL, Early efficient weight loss; EGWL, Early general weight loss. [file Image_1.tif]

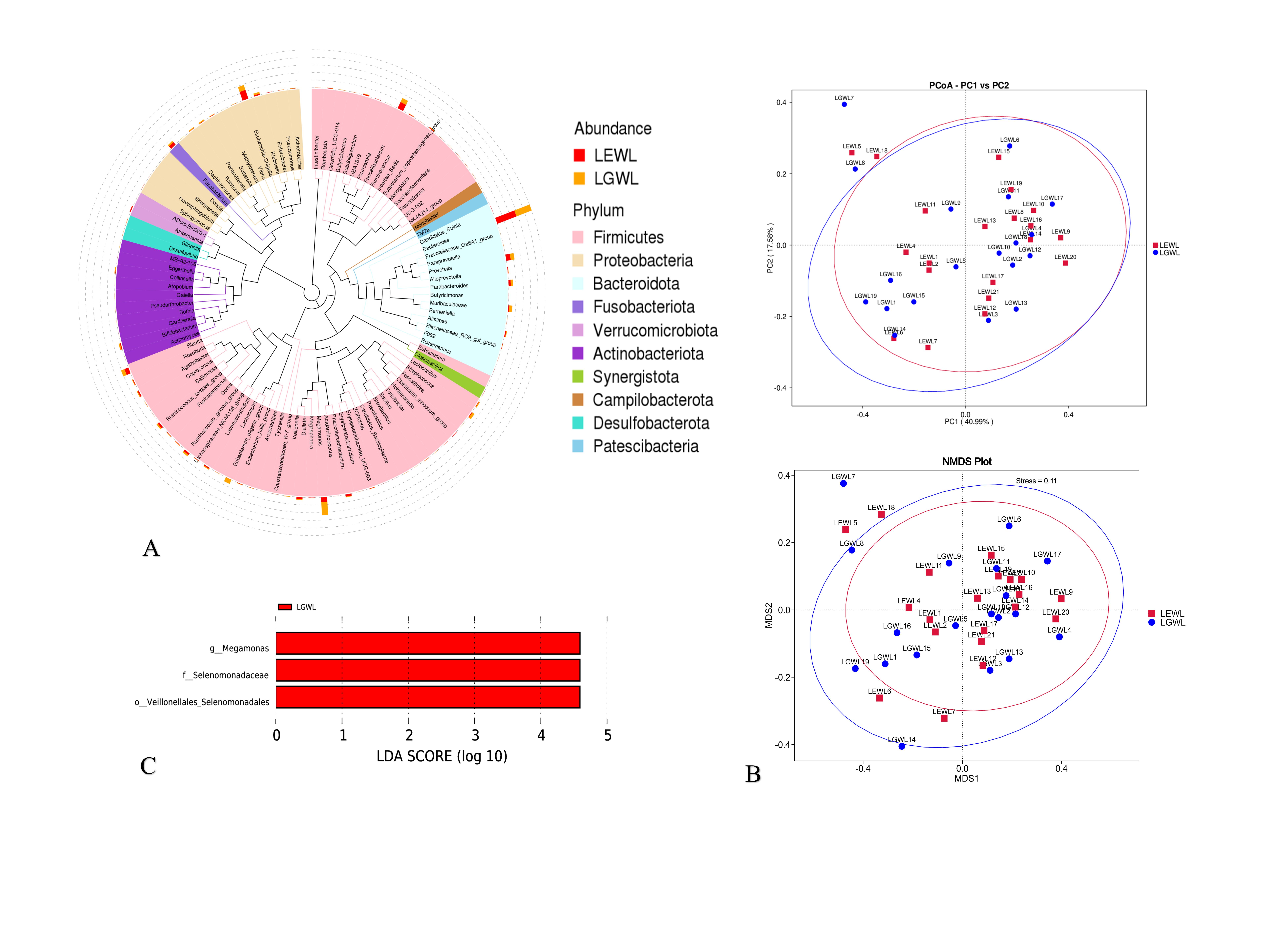

Supplement: SUPPLEMENTARY FIGURE 2 — The abundances of gut microbiota in the LEWL and LGWL groups. (A) The distribution of microbiota. (B) Beta diversity analysis by Co-ordinates Analysis (PCoA), Non-Metric Multi-Dimensional Scaling (NMDS). (C) LDA tree of LefSe analysis. LSG, Laparoscopic sleeve gastrectomy; LEWL, Later efficient weight loss; LGWL, Later general weight loss. [file Image_2.tif]

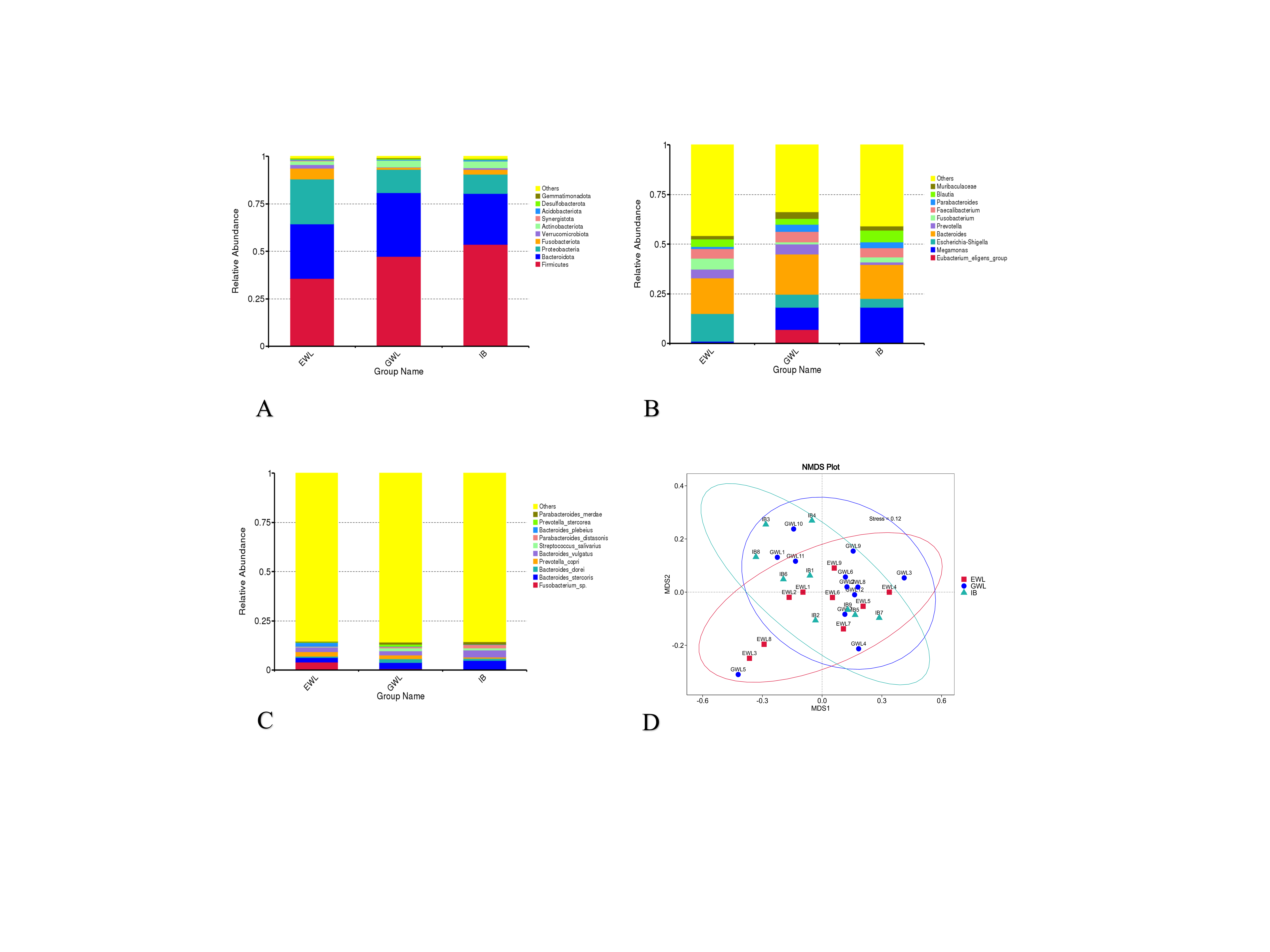

Supplement: SUPPLEMENTARY FIGURE 3 — The distribution in EWL, GWL and IB groups. (A–C) The distribution at phylum, genus and species levels in EWL, GWL and IB groups. (D) Beta diversity analysis by Non-Metric Multi-Dimensional Scaling (NMDS). LSG, Laparoscopic sleeve gastrectomy; EWL, efficient weight loss; GWL, general weight loss; IB, inconsistent effects between early and later stages. [file Image_3.tif]

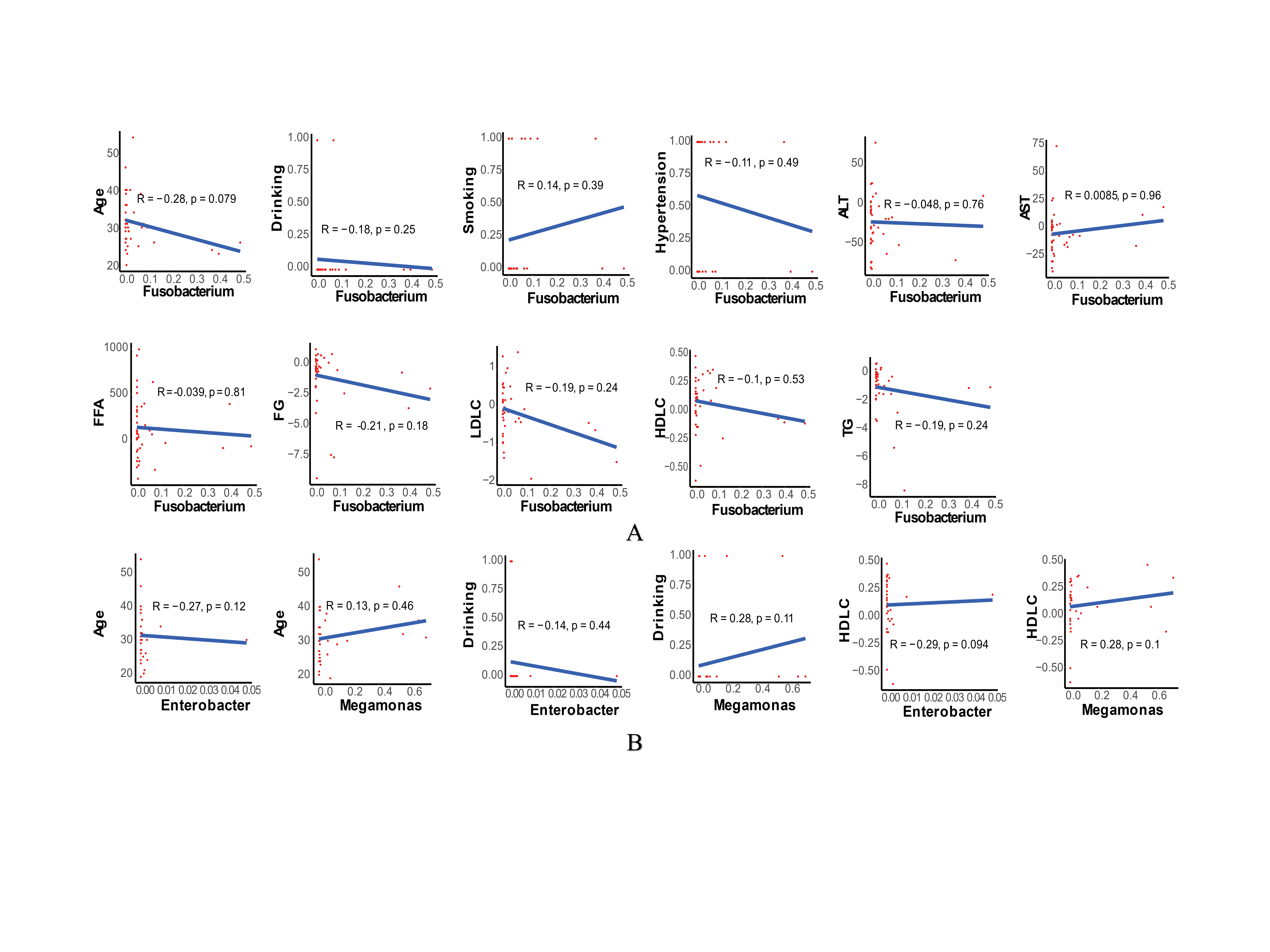

Supplement: SUPPLEMENTARY FIGURE 4 — Spearman correlation analysis. (A) Fusobacterium and clinical characteristics correlation analysis. (B) Enterobacter and Megamonas, and clinical characteristics correlation analysis. LSG, Laparoscopic sleeve gastrectomy; BMI, Body mass index. [file Image_4.tif]
