# Supplementary material for: The role of gut microbiota in predicting the weight loss following laparoscopic sleeve gastrectomy
Source: Front Microbiol. 2025 Mar 3;16:1560368. doi: 10.3389/fmicb.2025.1560368 (PMC11911518; doi:10.3389/fmicb.2025.1560368)
Supplement: Supplementary file 5 [file Table_1.docx]

| **Supplement Table 1. Alpha-diversity in the early and mid-term arms** | | | |
| --- | --- | --- | --- |
| Early Arm | | | |
|  | EEWL (n=25) | EGWL (n=18) | P value |
| Chao1 | 561.2 (382.4-844.5) | 346.2 (270.3-868.9) | 0.121 |
| Dominance | 0.054 (0.024-0.153) | 0.055 (0.335-0.152) | 0.739 |
| Shannon | 5.9 (4.5-6.7) | 5.8 (4.3-6.3) | 0.599 |
| Simpson | 0.95 (0.85-0.98) | 0.95 (0.85-0.97) | 0.739 |
| Later Arm | | | |
|  | LEWL (n=21) | LGWL (n=19) | P value |
| Chao1 | 551.7 (389.4-708.6) | 363.8 (265.9-975.4) | 0.301 |
| Dominance | 0.044 (0.029-0.067) | 0.043 (0.023-0.107) | 0.967 |
| Shannon | 6.1 (5.1-6.5) | 5.8 (4.7-7.0) | 0.835 |
| Simpson | 0.96 (0.93-0.97) | 0.95 (0.89-0.98) | 0.967 |
| Data were presented as medians with interquartile ranges. | | | |
